# 222 Creating a healthy culture through the promotion of physical activity in the university environment

**DOI:** 10.1093/eurpub/ckae114.010

**Published:** 2024-09-26

**Authors:** Alba Pardo-Fernández, Anna Castells, Irati Oliva

**Affiliations:** TecnoCampus Mataró-Maresme Pompeu Fabra University, Barcelona, Spain; High Performance and Health applied Technology Reseach Group (TAARS), TecnoCampus, Department of Health Sciences, Pompeu Fabra University, Barcelona, Spain; TecnoCampus Mataró-Maresme Pompeu Fabra University, Barcelona, Spain; High Performance and Health applied Technology Reseach Group (TAARS), TecnoCampus, Department of Health Sciences, Pompeu Fabra University, Barcelona, Spain; TecnoCampus Mataró-Maresme Pompeu Fabra University, Barcelona, Spain

## Abstract

**Purpose:**

Healthy Campus is a 7-year project to enhance health and lifestyle behaviours of the Tecnocampus community (Pompeu Fabra University). Healthy Campus includes a students’ commission and staff Health Ambassadors. Using the peer methodology in University community improves the level of Physical Activity (PA) habit acquisition and health.

**Methods:**

The strategic plan is evaluated annually using indicators such as activities’ participation and satisfaction. We implement a survey to assess health and lifestyles of the university community.

PA has been promoted mainly through the celebration of World PA Day (DMAF), being the first Catalan university certified in the promotion of bicycles, healthy messages in the building, creation of outdoor Urban health park and sports events. Volunteers students were trained as Health-Agents to co-design and promote different PA actions. PA promotion also has been included in academic curriculum.

In staff, activities of different exercise intensities have been promoted to satisfy different needs and preferences. Workers attend focus groups to detect needs and thus improve interventions in their colleagues.

**Results:**

In the 7 years of the project, 260 actions based on PA promotion has been developed. 165 volunteer students participated in the project and 25 staff health ambassadors participated in Focus-group. A total of 7,373 attended at conferences, courses or workshops. 2200 students participated in World PA Day. 700 staff participate in PA workshops, coursed and activities. 21 academic curricula subjects include PA promotion. This project work together with external entities such as City Council, sports clubs, senior residences, schools, hospitals.

**Conclusions:**

What greatly differentiates this project is the transversality, the peer methodology, the inclusion of PA promotion in academic curriculum and the PA promotion at multiple levels and environments: education, workplace and community level.

The creation of health ambassadors among equals empowers in a very relevant way the objectives of health promotion and PA. In conclusion, it is crucial to train students to be health-conscious citizens who in the future will have the opportunity to promote health in the general population and will influence their lifestyles. And also to create staff ambassadors who spread healthy PA habits.

**Support/Funding Source:**

Without financing

